# A Manual of Pathological Anatomy

**Published:** 1855-11

**Authors:** 


A Manual of Pathological Anatomy. By CARL ROKITANSKY, M. D., Curator of the Imperial Pathological Museum, and Professor at the University of Vienna, &c., &c. Volumes 1, 2, 3, & 4. Philadelphia: Blanchard & Lea, 1855.

THIS translation of Professor Rokitansky's celebrated work appeared under the auspices of the Sydenham Society. This fact of itself is a sufficient guaranty of the ability with which the task has been performed. The labor of the translation has been performed by four gentlemen, each well qualified for the work.

From the editor's preface to the first volume we extract the following notice of the author:

"Charles Rokitansky, the founder of the German [it should rather have been called Austrian] medico-anatomical school, was born at Koinigsgraetz, in Bohemia, was educated at the Gymnasium of Leitneritz, and graduated, at Vienna, in 1828. Shortly afterwards he was appoined Assistant in the pathologico-anatomical department of the University, and, in 1834, Professor of Pathological Anatomy. At the same time he was instituted Prosector at the General [united Civil and Military] Hospital at Vienna, and also sole medico-legal Anatomist for the examination of all doubtfnl cases of death throughout that metropolis.

The immense fund of materials thns placed at his disposal [the number of corpses dissected by him is summed up at 30,000] was almost entirely reserved for the elaboration of that grand work on pathological anatomy, which in the consciousness of having thoroughly mastered the subject, he gave to the world between the years 1842 and 1846; which has passed, *unaltered*, through three reimpressions; and which, under the auspices of the Sydenham Society, has been translated into the English language."

"In 1847, Rokitansky was appointed Dean of the Medical Faculty, and, in 1850, Rector of the University, of Vienna."

We cannot do justice to the author in the space and time allotted us for a book notice, but we wish to call attention to one or two points. In reference to cancer he says:

"The crasis which gives rise to the production of cancer, consists mainly in a preponderance of albumen, a defibrination (hypinosis), for the particulars of which we must refer to the doctrine of crasis. Concurrently with this we have, more especially in the medullary crasis, an excess of fat in the circulating fluid, which determinates a complication of cancer to be discussed in a more appropriate place; and, again, that remarkable relation of exclusiveness towards ordinary, fibrinous tubercle.

This crasis is essentially the same for all cancers, only exquisitely developed in the medullary form. This may be inferred, at least, from the frequent concurrence of various cancers species, in primative or consecutive combination, either in the same locality, or in different organs. It may also be inferred from the circumstances that, after extirpation, the one is replaced by the other under the same contingencies, and that, conforming with an augmentation of the crasis, the medullary cancer is generally the consecutive one, more especially where the substitution takes place rapidly.

The highest grades of cancer-crasis originate through infection, that is, through the reception into the lymphatics, or more especially into the bloodvessels, of cancer-cells, or of cancer-blastema, of a lax, soft, semi-fluid character. The blastema is carried thither by imbibition, partly in the mere act of nutrition, partly, with or without the cancer-cells, through the lymphatics of veins laid open by ulceration of the tumor, or lastly, by the cancer penetrating into the canals of bloodvessels. Injection thus brought about, occasions locally, or it may be remotely, both in large bloodvessels and in the capillaries, coagulations of blood. In the former case, these are cylindrical, branched, plug-like, or clavate coagula, adhearing to the internal bloodvessel membrane, or to the endocardium (vegetations). They reveal their cancerous nature by their external medullary characters, as well as by their vigorous growth. In the capillaries the coagulations assumes the form of the cancerous depot—so called metastasis (capillary phlebitis).

Cancer-formation assumes both the chronic and an acute course, the former being the more ordinary mode of occurrence for primitive cancer; whilst secondary cancer production is brought about with more and more rapidity in proportion as the cancers multiply. Ulceration and extripation of carcinoma are especially apt to determine its very acute secondary formation. Still there are instances of highly acute, primitive, general cancer production. Moreover, the individual species of cancer manifest marked differences in this respect, both the first development and the ulterior growth, for example, of fibrous cancer, being slow, whilst in the case of medullary cancer they are incomparably more rapid.

In primative cancers, the blastema is, in the great majority, insensibly produced. In acute cancer-formation it is thrown out under the symptoms of hyperæmia, and occasionally of inflammation. In the latter case, it often covers serious membranes with a stratiform cancer exudate, or infiltrates and hepatizes the lungs with cancerous tubercles. From what has been said, our opinion may be inferred respecting the seat of cancer, in opposition to that of Carswell and Cruvelhier, who refer its origin to the capillary system. But though in the ordinary process of cancer-formation we look upon the blastema as an exudate in its broadest sense, we by no means question the origin of cancer from coagulation within the bloodvessels after the type of depot-formation in general (see Metastasis). It is indeed to this mode of development that we would ascribe the rapid cancer-formation engendered, in brutes, by the injection of cancer-blastema.

We are further disposed—although from insolated facts only—to believe in cancer-formation, through a conversion of certain physiological elements into those of cancer. In the liver, namely, we occasionally light upon a process, limited to circumscribed patches, of pallescence and alteration of the parenchyma, with some augmentation of its volume. Upon further examination, the portion of liver so affected is found to consist indubitably of hepatic cells, more or less bereft of their biliary and coloring matter, and of an intermediate, whitish, albuminous blestema,—though the hepatic cell had become seemingly transformed into the cell of medullary carcinoma.

It will be seen from the above extract that the author believes in the essential unity of cancers of different varieties, the differences being mainly in the intensity and rapidity with which the producing causes act. The belief is also expressed that certain physiological elements may be converted into cancerous elements. This is not in accordance with the generally received doctrines on this subject. The microscopic elements of medullary carcinoma are thus described :—

(*a.*) Consisting of granulated cells with a more or less distinct nucleus, and resembling pus-globules.

(*b.*) Consisting of smaller and greater, granulated, round, or angular, protuberant cells, more or less resembling the cells of tessellated epithelium, the hepatic cells, the ganglion globules, and provided with one or several nuclei.

(*c.*) Consisting of spindle-shaped and caudate, nucleated cells, fibre-cells, amongst which are many others, both spherical and oval.

(*d.*) Consisting of elliptical corpuscles of 1-100 to 1-50 of a millimetre in circumference, and furnished with one or two nucleoli. They have the significance of a (heteroplastic) transcendent development of cell nuclei.

(*e.*) Consisting of spherical or oval corpuscles corresponding in size and tendency with the cell nucleus.

(*f.*) Consisting of elementary granules down to the finest molecule-mass, with scanty nucleus formations in progress of development.

(*g.*) A further element concurrent with those specified at *b*, are pouch-like formations (see Metamorphosis of Blastema), and chiefly the parent-cell, which often constitutes a prominent element in medullary cancers. It forms here again the groundwork for the alveolar textural type of medullary cancer.

These elements occur predominantly, it may be, in the one or the other form, but intermingled with others. Viewed with the naked eye, the elementary composition of a texure is, even to the well initiated, a matter rather of conjecture than of any certainty. The consistency and density of a texture may vary infinitely, being dependent upon the character of the intercellular substance. It is only where there is the appearance of fibrillation that we may perhaps infer a composition of spindle-shaped or caudate cells.

Differences more important affect the character of the intercellular substance, and of a stroma in which the elements adverted to lie imbedded. This stroma in which the elements themselves, which according to the laws of the cell theory, form into a fibrous skeleton work ; or else it springs immediately out of a consolidated, amorphous, intercellular substance. Both together occasion, in medullary carcinoma, a special structure manifest to the naked eye, in the shape of a variously disposed fibrillation and lobulation, &c., the character of which so greatly modifies the consistency of the heterologous growth.

In this regard, we have the following forms, some more or less cognizable with the naked eye.

(*a.*) A medullary carcinoma, with an amorphous fluid, or semifluid, intercellular substance. The aforesaid elements vegetate in a thin or a thickish medullary juice. It is represented in the very lax, milky or cream-like *encephaloid* cancer.

(*b.*) A medullary carcinoma, with a solidified, amorphous, or else striated, indefinitely fibrous, intercellular substance, interspersed with roundish and fibro-elongated nuclei.

(*c.*) Medullary carcinoma, with a stroma consisting of fibre-cells (spindle-shaped, caudate) arising out of the developement of the elements of the medullary substance itself, with consumption of the intercellular substance, and condensation of the heterologous growth.

(*d.*) Medullary cancer with a delicate hyaline, structureless, or else an opaque, striated, membranous stroma, studded with elementary granules and nucleus formations, or fibrillated like areolar tissue; which stroma, at the same time, forms the groundwork for the vascularization of the alien growth. Its interspaces are filled with a loose, fluid medullary matter, and it is easily thrown into relief if the tumor be scraped, pressed, or simply steeped in water. In villous cancer this stroma appears developed into a main constitutent.

(*e.*) Medullary carcinoma with a more or less developed fibrous stroma, whose fibre-elements, upspringing from a solidified blastema, now resemble fibre-cellular tissue, now organic muscle-fibre. It represents either a scaffold-work or a stellate structure, the gaps being filled up with embryonic elements. Even with the naked eye it is discernible as denser striæ, disposed as aforesaid, and remarkable for their whiteness and their tendon-like lustre. This stroma has frequently the significance of fibrous cancer blended with medullary. It is, however, often enough an innocent fibroid growth, which may very possibly become the seat of so-called ossification (bony concretion). Hence the extraordinary phenomenon of medullary cancer becoming traversed by a concrete skeleton-work, in the midst even of soft parts.

The author speaks of the cure of cancer, "by the progressive destruction, necrosis and partial rejection of the tumor, or else by its more rapid death and expulsion, a circumscribing supuration isolating it from the healthy textures."

"Saponification" and "decadency" or "wasting of the tumor" are enumerated as terminations of the disease.

The author commences the chapter on blood disease by remarking that "humeral pathology is simply a requirement of common practical sense."

The anomalies of blastema constitute a favorite theme with Prof R., and upon them he bases the doctrine of new formations both malignant and non, malignant, exudations and outgrowths of whatever kind, are all referred to some peculiar crasis or special constitution of the blood. The pathological anatomist, however, can accomplish only half of the work of detecting these different crasis. To the chemical Pathologist we are under quite as strong obligations.

The second volume is translated by Dr. Edward Sieveking, whose labors in pathological anatomy are already familiar to the profession.

We have only room for a single quotation on the pathology of "Bright's disease."

We consider the nature of Bright's disease to consist in an inflammatory process, which proceeds from a stage of hyperæmia to one of stasis, and then gives rise to a product, which is not only remarkable by its peculiar character, but which, in well-marked cases, by its excessive accumulation, causes a singlar alteration in the appearance and structure of the kidney. It commonly runs, as we have already stated, a chronic course, with occasional exacerbations, but it is sometimes acute. In the latter very important case, in which, from the tumultuous violence of the exudation, the product is mixed with a large amout of serum, and is generally reddened by the coloring matter of the blood, and in which the characteristic milky or creamy or coagulated substance of well-marked Bright's disease is not formed, we should be obliged to consider the condition as one of very acute simple inflammation of the kidneys, were it not that the characteristic general symptoms and the constitution of the urine established it as a case of Bright's disease.

The whitish or ashy, milky or creamy product, which may resemble albumen in its degrees of coagulation, and consists of solitary and accumulated molecules, or of a more or less globular fibrinous coagula and pus-corpuscles (Gluge), is an albuminofibrinous substance, with a predominance of albumen; the amount in which it occurs is proportioned to the amount of granular degeneration.

The product may, as in simple inflammation, be deposited at every point of the renal parenchyma external to the vessels, but we find it more particularly in the Malpighian bodies (glands), and subsequently in the urinary tubuli; the granulations of Bright's disease are therefore in reality the Malpighian corpuscles charged with the above-named substance. The more the latter accumulates, the more it interferes with the circulation, hence the peculiar pallor or anæmic condition of the organ.

The cause of peculiar character of the product is the more obscure, since the question is generally evaded. As the amount of reaction that takes place in the renal tissue does not suffice to explain it, we are led to seek the cause in an anomalous constitution of the blood, consisting in an excess of albumen, which may originate in a decomposition of the fibrine. This becomes the more probable, when we consider that the most frequent exciting cause (cold) appears peculiarly adapted to give rise rather to a change in the blood, than to a disease to the kidneys, and that the infiltration of the kidney, which we have examined as the eight form, is evidently developed as a sequel of the cachexiæ which we shall shortly investigate, and in complication with similar affections of other organs (liver, spleen). Although we might offer numerous observations on this connection, the real cause of the development of the renal disease from the crasis of the blood, which often takes place with such extreme rapidity, is to us an enigma. We look upon the anomolous condition of the blood in Bright's disease as the primary affection, which, from a peculiar relation to the kidneys, is followed by the secondary and visible disorganization of the renal tissue; this need not however always ensue; at all events t des not follow as rapidly as the structural disease of the kidney consequent upon the vegetative disturbance that causes diabates mellitus. By this means we explain how it happens that the two kidneys are generally attacked at the same time or at brief intervals. Graves is of opinion that the change of texture is induced by free acids of the urine (phosphoric and nitric acids) coagulating the albumen as it passes into the urinary tubuli.

The author seems to regard the albumen as pathognomonic of the disease, for he speaks of the liability to mistake the disease for very acute simple inflammation, were it not for the characteristic general symptoms and the constitution of the urine. He also speaks of the tendency to Bright's disease after scarlatina, and the cure by resolution.

We have not time to follow the author further—at some future day we may refer to the second and third volumes.

The reputation of the author and the auspices under which it appears, renders it unnecessary for us to say one word in commendation of the work.

For sale by D. B. Cooke & Co., Chicago, Illinois. J.

